# Diagnostic performance in T staging for patients with esophagogastric junction cancer using high-resolution MRI: a comparison with conventional MRI at 3 tesla

**DOI:** 10.1186/s40644-019-0269-6

**Published:** 2019-12-04

**Authors:** Yuan Yuan, Luguang Chen, Shengnan Ren, Zhen Wang, Yukun Chen, Aiguo Jin, Shuai Li, Xu Fang, Tiegong Wang, Yun Bian, Qingsong Yang, Chenguang Bai, Qiang Hao, Jianping Lu

**Affiliations:** 1 Department of Radiology, Changhai Hospital of Shanghai, Second Military Medical University, No.168 Changhai Road, Shanghai, 200433 China; 2 Department of Nuclear medicine, Changhai Hospital of Shanghai, Shanghai, China; 3 Department of Pathology, Changhai Hospital of Shanghai, Shanghai, China

**Keywords:** Esophagogastric junction cancer, T staging, High-resolution MRI

## Abstract

**Background:**

To investigate and compare the diagnostic performance in T staging for patients with esophagogastric junction cancer using high-resolution magnetic resonance imaging (HR MRI), as compared with conventional MRI at 3 Tesla.

**Methods:**

A total of 118 patients with pathologically confirmed esophagogastric junction cancer were included and underwent multiparameter HR MRI (Cohort 1, 62 patients) or conventional MRI (Cohort 2, 56 patients). T2-weighted, T1-weighted, diffusion-weighted and contrast-enhanced T1-weighted images of each patient were evaluated by two radiologists who determined the preoperative T staging by consensus. Using pathologic staging as the gold standard, the consistency between HR MRI and pathology and between conventional MRI and pathology in T staging was calculated and compared. The overall accuracy, overstatement and understatement of HR MRI and conventional MRI in T staging of patients with esophagogastric junction cancer were computed and compared. Moreover, the diagnostic performance of HR MRI and conventional MRI in T staging (≤ T1 and ≥ T4) of patients with esophagogastric junction cancer were evaluated.

**Results:**

There were no significant differences in age (*p* = 0.465) and sex (*p* = 0.175) between Cohorts 1 and 2. Excellent agreement was observed in the T staging of patients with esophagogastric junction cancer between pathology and HR MRI (kappa = 0.813), while moderate agreement was observed between pathology and conventional MRI (kappa = 0.486). Significant differences were observed in overall accuracy (88.7% vs 64.3%, *p* = 0.002) and understatement (1.6% vs 26.8%, *p* < 0.001) but not for overstatement (9.7% vs 8.9%, *p* = 0.889) in T staging between HR MRI and conventional MRI techniques. For differentiating the T stages of ≤ T1 from ≥ T2 and the T stages of ≤ T3 from ≥ T4, no significant differences were observed between the imaging techniques.

**Conclusions:**

HR MRI has good diagnostic performance and may serve as an alternative technique in the T staging of patients with esophagogastric junction cancer in clinical practice.

## Background

The prevalence of esophagogastric junction cancer has increased in Western countries and Japan in recent decades [1–3]. The T staging of esophagogastric cancer is important for making treatment plans and is a core component of the TNM staging system. Endoscopic therapy is an option for patients with T1 stage disease. Patients with T2-T4a stage cancer may have a chance of undergoing radical surgery and may need neoadjuvant chemoradiotherapy before surgery, depending on the stage. Patients with T4b are not eligible for undergoing surgery [4]. Therefore, an accurate and efficient method is needed to differentiate these stages and to provide a treatment reference for clinicians.

In recent years, several techniques have been used to evaluate gastric cancer, such as endoscopic ultrasonography (EUS), multidetector spiral CT (MDCT), positron emission tomography/computed tomography (PET/CT), and magnetic resonance imaging (MRI) [5–7]. Ultrasound endoscopy has moderate accuracy in T staging, but it requires the administration of sedative agent and depends on the operator’s experience [6]. MDCT is the most commonly used screening tool because of its short scan time, high spatial resolution, and low cost; however, it exposes patients to ionizing radiation and has a risk of causing a contrast agent allergic reaction [5]. PET/CT is a whole-body and systemic imaging technique that has great value for detecting distant metastasis of tumors, but is expensive and uses ionizing radiation [5, 7]. MRI is a noninvasive imaging technique with advantages that include no ionizing radiation, multiple parameters, arbitrary planar imaging, low probability of contrast agent allergic reaction, and high soft tissue resolution [5]. In addition, improved MRI technologies can reduce scan time and improve image quality, making MRI a good technique for clinically assessing esophagogastric junction cancer [8–10].

Several studies have reported that ex vivo high-resolution MRI (HR MRI) can be used to delineate 4-layer (e.g., mucosal layer, mucosal muscle layer, submucosa, and muscularis propria) or even 7-layer structures in the stomach wall. Those reports found that HR MRI was highly accurate for evaluating the invasion depth of gastric cancer [11–13]. HR MRI (thin slice thickness, small field of view or large matrix) has been used to evaluate the preoperative staging of tumors in clinical practice, such as esophagus cancer and rectal cancer; in addition, it offers high spatial resolution and good image contrast and has a great potential for assessing the infiltration depth of esophagogastric junction lesions [14–20]. However, the evaluation of patients with esophagogastric junction lesions using the HR MRI technique has not been explored thus far.

Therefore, the purpose of the present study was to investigate and compare the diagnostic performance in T staging for patients with esophagogastric junction cancer using HR MRI and conventional MRI at 3 Tesla.

## Methods

### Patients

This retrospective study was approved by the local institutional review board, and written informed consent was waived for each patient. Between January 2017 and December 2018, 118 patients who underwent multiparameter MRI examination were evaluated (Cohort 1: 62 patients were scanned using HR MRI techniques; Cohort 2: 56 patients were scanned using conventional MRI techniques). All patients with esophagogastric junction cancer were confirmed by gastroscopy and biopsy. Both cohorts used the same inclusion and exclusion criteria. Patients were included if they met the following criteria: (1) gastroscopic biopsy confirmed esophagogastric junction cancer, (2) underwent radical surgery or abdominal exploration, (3) no chemoradiotherapy treatment prior to operation, and (4) time interval between MRI and surgical operation was within one week. Patients were excluded for any of the following reasons: (1) preoperative imaging findings revealed distant metastases, such as liver metastasis and retroperitoneal lymph node metastasis; (2) image artifacts due to patients with poor cooperation; (3) MRI contraindications, such as cardiac pacemaker implantation, unknown metal material in the body, claustrophobia, renal insufficiency, or a history of allergic reactions to the gadolinium contrast agent. All patients were fasted for at least 5 h, and warm water (800 ml) was administered to dilate the stomach before the MRI examination.

### Magnetic resonance imaging

All MRI scans were performed on a 3 Tesla MRI scanner (MAGNETOM Skyra, Siemens Healthcare, Erlangen, Germany); an 18-channel phased-array body and integrated spine coils were used to receive the MR signal. High-resolution and conventional multicontrast transverse MRI protocols were used to evaluate the lesions in patients from Cohorts 1 and 2, respectively, and included the following sequences: two-dimensional T2-weighted turbo spin echo (2D T2W TSE) with motion-insensitive (BLADE) and respiratory triggering to minimize motion artifacts, diffusion-weighted imaging (DWI) was performed with the respiratory-triggered single-shot spin-echo echo-planar technique, three-dimensional T1-weighted volume interpolated body examination (3D T1W VIBE) and three contrast-enhanced 3D T1W VIBE imaging phases (arterial, venous and delayed phases). The main imaging parameters are listed in Table 1. Contrast-enhanced T1W images were acquired at 30, 60 and 90 s after contrast administration, which consisted of 0.2 ml/kilogram body weight Gd-DTPA (Beilu, Beijing, China) delivered using an automatic power injector (Medrad Spectris Solaris EP MR Injector System, PA, USA) at 2 ml/s followed by a 20 ml saline flush at the same rate.

**Table 1:** The main imaging parameters of MRI protocols

	Protocols	TR/TE (ms)	FOV (mm^2^)	Matrix	FA(°)	ST (mm)	Gap	Slices	FS	Averages	TA
HR MRI, Cohort 1	T2 W	6500/87	260*260	256*256	131	3	0.6	30	No	1	3.5–4.5 min
	DWI^a^	2000/51	195*260	180*240	90	3	0.6	30	Yes	–	4–5 min
	T1W	4.23/1.13	175*280	160*256	12	3	0	28	Yes	3	16 s
	T1W + C^c^	4.23/1/13	175*280	160*256	12	3	0	28	Yes	3	16 s
Conventional MRI, Cohort 2	T2 W	4560/79	380*380	320*320	140	6	1.2	28	Yes	1	3–4 min
	DWI^b^	2200/55	296*395	96*128	90	6	1.2	20	Yes	–	31 s
	T1W	3.97/1.26	325*400	195*320	9	3	0	64	Yes	1	15 s
	T1W + C^c^	3.46/1.32	308*380	195*320	12	3	0	64	Yes	1	14 s

*FA* flip angle, *ST* slice thickness, *FS* fat saturation, *TA* acquisition time

^a^b values (number of averages), 50 (1), 100 (1), 150 (1), 200 (1), 500 (2), 800 (3) s/mm^2^

^b^b values (Number of averages), 50 (1), 800 (3) s/mm^2^

^c^arterial, venous and delayed phases

### Image analysis

All images were transferred to an advanced workstation for further analysis. For Cohorts 1 and 2, a similar MR image set was assessed for each patient, which included T2WI, DWI, T1WI and 3 imaging phases of contrast-enhanced T1WI, with the procedures performed using different imaging techniques (high-resolution and conventional MRI protocols for Cohorts 1 and 2, respectively). Two experienced radiologists (Y. Y. and S. L. with 9 and 7 years of experience in diagnostic radiology, respectively) randomly evaluated all the patient image sets by consensus; however, they were blinded to all clinical information for each patient. All MR images were evaluated by the following T staging criteria: ≤ T1 (Tis, T1a and T1b), no lesions found or a focal thickening of the inner layer of the gastric wall; T2, partial thickening of the gastric wall with a smooth, well-defined outer border; T3, complete thickening of the gastric wall with a smooth, well-defined outer border; and T4, complete thickening of the gastric wall with an unsmoothed outer border or invasion of adjacent organs [21].

### Pathological evaluation

The tumors were staged based on the histopathological findings for assigning the tumor stage according to the TNM staging, as described by the American Joint Committee on Cancer (AJCC, 8th edition) [22]. Histopathological analysis of the resected specimens was performed by a pathologist (C. B., who has 15 years of experience in diagnosing gastrointestinal lesions) with a special emphasis on the invasion depth, degree of differentiation, Sievert type, and other common findings [23].

### Statistical analysis

SPSS software (version 20.0, Inc., Chicago, IL, USA) was used to perform statistical analyses. Continuous variables are presented as the mean ± standard deviation, and categorical variables are expressed as percentages. Differences in age and sex between patients in Cohorts 1 and 2 were tested using independent sample t-tests and chi-squared tests, respectively. Using the pathologic staging results as the gold standard, the consistency in the T staging between HR MRI and pathology and between conventional MRI and pathology was calculated and compared by the chi-squared test. According to Landis and Koch, kappa values > 0.8 indicate excellent agreement, 0.6 to 0.8 indicate substantial agreement, 0.4 to 0.6 indicate moderate agreement, 0.2 to 0.4 indicate fair agreement, 0.0 to 0.2 indicate slight agreement, and < 0.0 indicate poor agreement [24]. The overall accuracy, overstatement and understatement of HR MRI and conventional MRI in the T staging of patients with esophagogastric junction cancer were computed and compared by chi-squared test. Moreover, the diagnostic performance of HR MRI and conventional MRI in T staging of patients with esophagogastric junction cancer was evaluated for the T staging of ≤ T3 from ≥ T4 and of ≤ T1 from ≥ T2. A *p* value < 0.05 indicated statistical significance.

## Results

### Patient demographics

All patients underwent surgeries, and each patient had 1 identified lesion. In Cohort 1, 62 patients were enrolled and underwent HR MRI examinations; these patients included 47 males and 15 females with a mean age of 64.2 ± 10.8 years old. In Cohort 2, 56 patients were included and underwent conventional MRI examinations; these patients included 48 males and 8 females with a mean age of 62.8 ± 10.8 years old. The details of patient demographics are presented in Table 2. There were no significant differences in age (*p* = 0.465) and sex (*p* = 0.175) between Cohorts 1 and 2.

**Table 2:** Patient demographics

Variable	Cohort 1 (*n* = 62, %)	Cohort 2 (*n* = 56, %)
Gender		
Male	47 (75.8)	48 (85.7)
Female	15 (24.2)	8 (14.3)
Age (years)		
Mean ± SD	64.2 ± 10.8	62.8 ± 10.8
Pathological differentiation		
Poor	11 (17.7)	17 (30.4)
Moderate to poor	22 (35.5)	15 (26.8)
Moderate	26 (41.9)	20 (35.7)
High to moderate	2 (3.2)	1 (1.8)
High	1 (1.6)	3 (5.4)
T stage		
≤ T1	10 (16.1)	11 (19.6)
T2	7 (11.3)	4 (7.1)
T3	36 (58.1)	32 (57.1)
T4a	7 (11.3)	9 (16.1)
T4b	2 (3.2)	0 (0.0)
Siewert type		
I	0 (0.0)	0 (0.0)
II	44 (71.0)	32 (57.1)
III	18 (29.0)	24 (42.9)

≤T1: Tis, T1a and T1b

### Diagnostic agreement in the T staging

**Fig. 1:**
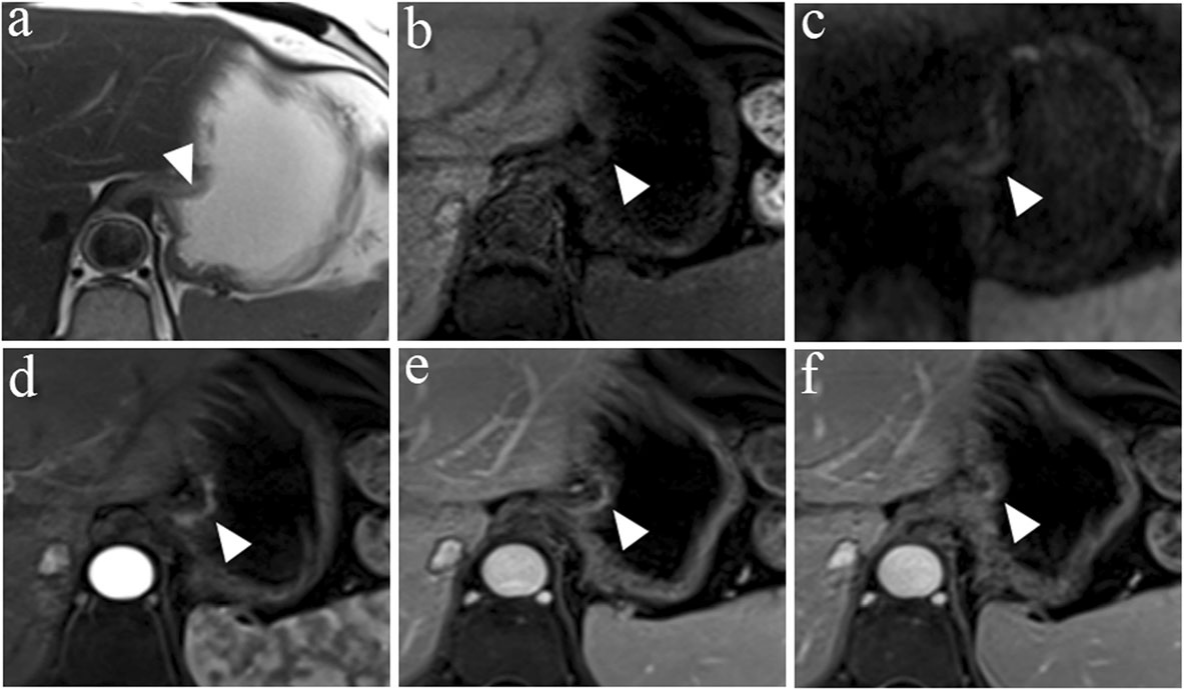
A 68-year-old woman with esophagogastric junction cancer was pathologically diagnosed using HR MRI as having stage T1b, Sierwert II. **a** The axial T2W image shows partial thickening of the cardia wall with a decreased signal (arrow). **b** The axial diffusion-weighted image with b = 800 s/mm^2^ shows restricted diffusion in the cardia. **c** The lesion was not clearly shown on the axial T1W image. **d** Axial and arterial phases of a contrast-enhanced image show significant enhancement of the lesion (arrow). **e** Persistent enhancement of the lesion was observed on the enhanced and venous phases of T1W images. **f** The lesion and mucosal tissue had similar degrees of enhancement on the delayed phase of the T1W image

**Fig. 2:**
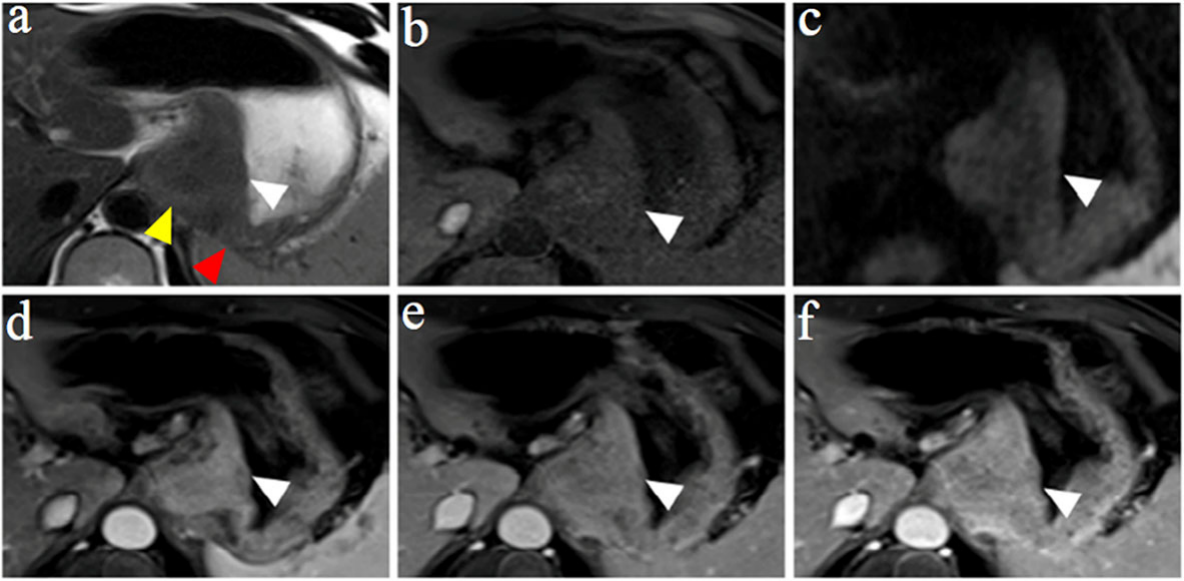
A 48-year-old man with esophagogastric junction cancer was pathologically diagnosed using HR MRI as having stage T4b, Sierwert II. **a** The axial T2W image shows clear thickening of the cardia wall, and the invasion of the left diaphragmatic crus and spleen was also observed (white, yellow and red arrows, respectively). **b** The axial diffusion-weighted image with b = 800 s/mm^2^ showing hyperintensity in the cardia wall. **c** An inhomogeneous thickening pattern of the cardia wall is shown on the axial T1W image. **d**–**f** The lesion was markedly enhanced and invaded into the left diaphragmatic crus and spleen on arterial, venous and delayed phases of the enhanced T1W images

**Fig. 3:**
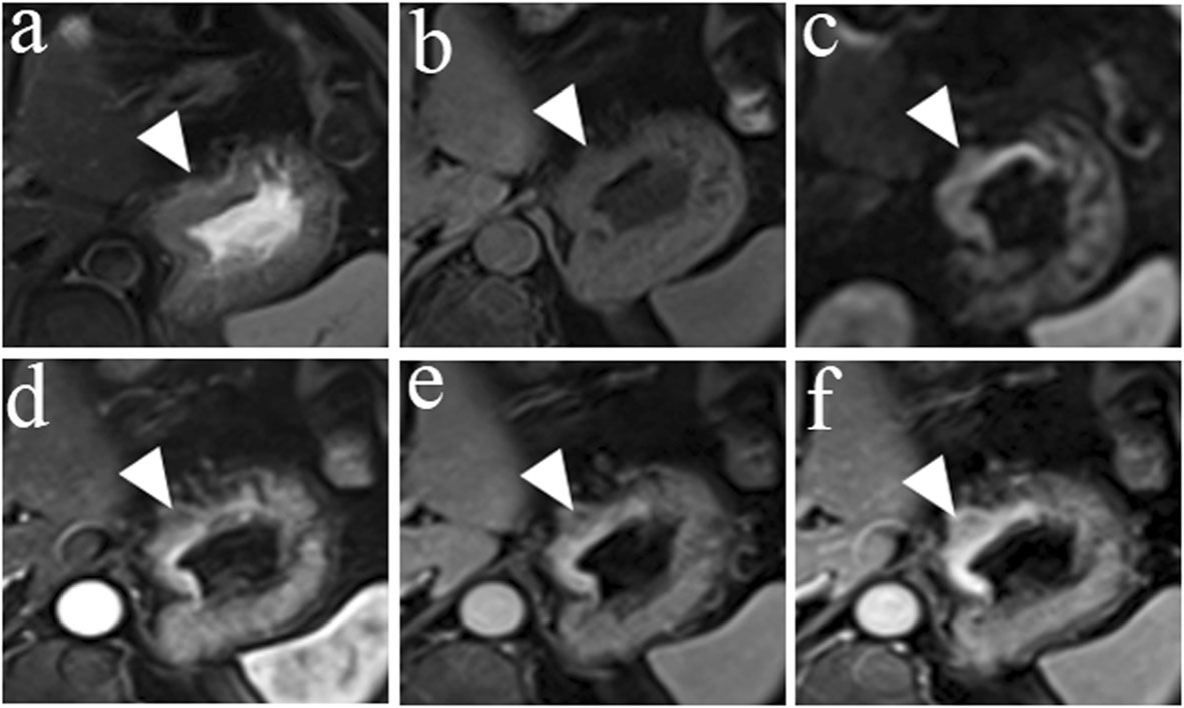
A 68-year-old man with esophagogastric junction cancer was pathologically diagnosed using conventional MRI as having stage T3, Sierwert II. **a** The axial T2W image shows the thickening and slight hyperintensity of the cardia wall and the peripheral adipose tissue was clear. **b** The axial diffusion-weighted image with b = 800 s/mm^2^ shows hyperintensity in the cardia wall. **c** A thickening pattern in the cardia wall is shown on an axial T1W image. **d**–**f** Compared with the adjacent normal tissues, the lesion was markedly enhanced on the arterial, venous and delayed phases of enhanced T1W images

**Fig. 4:**
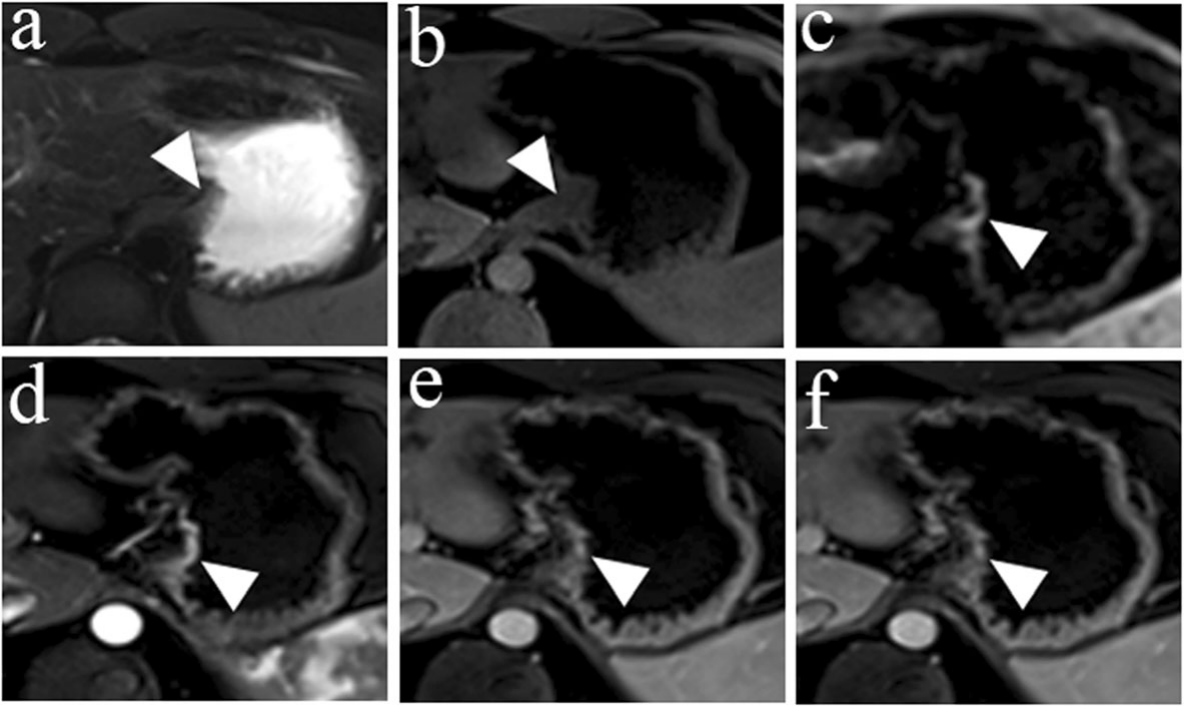
A 24-year-old man with esophagogastric junction cancer pathologically diagnosed as stage T1a, Sierwert III and was performed using conventional MRI. **a** The axial T2W image shows a lack of obvious thickening of the cardia wall. **b** The axial diffusion-weighted image with b = 800 s/mm^2^ shows hyperintensity in the cardia wall. **c** No thickening pattern of the cardia wall was shown in the axial T1W image. **d** The arterial phase of the enhanced T1W image shows a marked enhancement of the lesion. **e**, **f** Compared with the adjacent normal tissues, the lesion showed a similar enhanced pattern on venous and delayed phases of enhanced T1W images

Table 3 shows the diagnostic agreement between pathology and HR MRI and between pathology and conventional MRI for the T staging of patients with esophagogastric junction cancer. There was excellent agreement between pathology and HR MRI for the T staging of patients with esophagogastric junction cancer, with a kappa value = 0.813. However, only a moderate agreement was observed between pathology and conventional MRI, with a kappa value = 0.486. Representative images are shown in Figs. 1, 2, 3, and 4.

**Table 3:** The diagnostic results of HR MRI and conventional MRI in T staging of patients with esophagogastric junction cancer

Pathology	HR MRI^a^				Conventional MRI^b^			
	T1	T2	T3	T4	T1	T2	T3	T4
T1	9	1	0	0	11	0	0	0
T2	0	5	2	0	0	3	1	0
T3	0	0	33	3	3	7	18	4
T4	0	0	1	8	1	2	2	4

^a^Kappa = 0.813, *p* < 0.0001

^b^Kappa = 0.486, *p* < 0.0001

*HR MRI* High-resolution magnetic resonance imaging

### Comparison of the overall accuracy and diagnostic performance of HR MRI and conventional MRI in the T staging

In Cohort 1, 1 patient with T1, 2 patients with T2 and 3 patients with T3 were overstated, and 1 patient with T4 was understated. The overall accuracy, overstatement and understatement in the T staging of patients with esophagogastric junction cancer according to HR MRI were 88.7% (55/62), 9.7% (6/62) and 1.6% (1/62), respectively (Table 4). In Cohort 2, 1 patient with T2 and 4 patients with T3 were overstated, while 10 patients with T3 and 5 patients with T4 were understated. The overall accuracy, overstatement and understatement in T staging of patients with esophagogastric junction cancer according to conventional MRI were 64.3% (36/56), 8.9% (5/56) and 26.8% (15/56), respectively. There were significant differences in the overall accuracy (88.7% vs 64.3%, *p* = 0.002) and understatement (1.6% vs 26.8%, *p* < 0.001) between HR MRI and conventional MRI techniques in the T staging of patients with esophagogastric junction cancer, but there was no significant difference in overstatement (9.7% vs 8.9%, *p* = 0.889) in the T staging (Table 4).

**Table 4:** The accuracy, overstatement and understatement of HR MRI and conventional MRI in T staging of patients with esophagogastric junction cancer

T staging	HR MRI			Conventional MRI		
	Accuracy (%)	Overstatement (%)	Understatement (%)	Accuracy (%)	Overstatement (%)	Understatement (%)
≤T1	90.0 (9/10)	10 (1/10)	0 (0/10)	100.0 (11/11)	0 (0/11)	0 (0/11)
T2	71.4 (5/7)	28.6 (2/7)	0 (0/7)	75.0 (3/4)	25.0 (1/4)	0 (0/4)
T3	91.7 (33/36)	8.3 (3/36)	0 (0/36)	56.3 (18/32)	12.5 (4/32)	31.3 (10/32)
T4	88.9 (8/9)	0 (0/9)	11.1 (1/9)	44.4 (4/9)	0 (0/9)	55.6 (5/9)
Total	88.7 (55/62)^a^	9.7 (6/62)^b^	1.6 (1/62)^c^	64.3 (36/56)^a^	8.9 (5/56)^b^	26.8 (15/56)^c^

*HR MRI* High-resolution magnetic resonance imaging

^a^*p* = 0.002

^b^*p* = 0.889

^c^*p* = 0.000

Table 5 shows the diagnostic performance of HR MRI and conventional MRI techniques in the T staging of patients with esophagogastric junction cancer. For differentiating the T stages of ≤ T3 from ≥ T4, there were no significant differences in accuracy, sensitivity, specificity, overstatement and understatement between HR MRI and conventional MRI techniques (93.5% vs 83.9, 94.3% vs 91.5, 88.9% vs 44.4, 4.8% vs 7.1, 1.6% vs 8.9%, respectively, all *p* > 0.05). For differentiating the T stage of ≤ T1 from ≥ T2, none of the parameters indicated any significant differences between the two imaging techniques (all *p* > 0.05).

**Table 5:** Diagnostic performance of HR MRI and conventional MRI in T staging of patients with esophagogastric junction cancer

T staging	Technique	Accuracy (%)	Sensitivity (%)	Specificity (%)	Overstatement (%)	Understatement (%)
≤T3 vs ≥ T4	HRMRI	93.5 (58/62)	94.3 (50/53)	88.9 (8/9)	4.8 (3/62)	1.6 (1/62)
	ConventionalMRI	83.9 (47/56)	91.5 (43/47)	44.4 (4/9)	7.1 (4/56)	8.9 (5/56)
*p*		0.096	0.869	0.134	0.890	0.166
≤T1 vs ≥ T2	HR MRI	98.4 (61/62)	90 (9/10)	100 (52/52)	1.6 (1/62)	0 (0/62)
	ConventionalMRI	92.9 (52/56)	100 (11/11)	91.1 (41/45)	0 (0/56)	7.1 (4/56)
*p*		0.302	0.476	0.092	1.000	0.103

*HR MRI* High-resolution magnetic resonance imaging

## Discussion

Our study shows that when pathological staging was used as the gold standard, HR MRI had an excellent agreement in assessing the T stage among patients with esophagogastric junction cancer, while only a moderate agreement was observed for T staging achieved using conventional MRI. In addition, there were significant differences in the overall accuracy and understatement between the two imaging techniques.

According to various studies, the overall accuracy rate in evaluating the T stage of patients with gastric cancer using MRI ranges from 73.3 to 88.2%, with one study showing that the overestimation and underestimation rates were 6.7 and 20%, respectively [25–29]. The overall accuracy rate in T staging of patients with esophageal cancer by MRI was 69.8–90.9% [14, 17, 29]. Compared with patients with esophageal cancer or gastric cancer diagnosed using MRI, few studies have explored T staging in patients with esophagogastric junction cancer using MRI because of its special anatomical location. The current study found that the overall accuracy rate of T staging using HR MRI was high in comparison with that reported in the literature and was significantly higher than that achieved using conventional MRI. The significant improvement in the accuracy rate depended on the improved image resolution and contrast, which were of great significance in assessing the infiltration depth of the lesions, especially when distinguishing T3 from T4a [16, 30]. There was no significant difference in the overestimation rate between the two techniques, which is similar to reports in the literature. A significant difference was observed in the underestimation rate between the techniques. The underestimation rate in the T staging determined using conventional MRI was similar to that reported in the literature, while the T stages determined using HR MRI were lower than those previously reported [27]. This was because HR MRI can clearly delineate the scope and depth of the lesion. Only one patient diagnosed with T4a using HR MRI was underestimated as T3. The main reason may be the moderate enhancement of the lesion and the limited image contrast between the lesion and surrounding normal tissues. Fifteen patients had underestimated T stages when using conventional MRI; moreover, 1 patient with T4a and 2 patients with T3 were directly degraded to ≤ T1 because the scopes of the lesions were not fully displayed on conventional MRI, which did not accurately capture the boundaries of the lesions. This may also have been related to insufficient water filling in the stomach [31]. For T1b of esophagogastric junction cancer, the MRI findings generally showed linear enhancement. Some T1a lesions showed localized enhancement with a smaller area than that of T1b lesions, while some lesions could not be displayed. These enhancement patterns were similar to those reported by Lee et al. [32].

Our study showed that HR MRI has significantly higher soft tissue resolution than conventional MRI and was accurate and complete in displaying the location, extent and depth of invasion of the lesions. One study found that high-resolution T2WI is superior to conventional T2WI in displaying peripheral adipose tissue [20]. When other parameters remained unchanged, the reduced FOV naturally amplified the display of peripheral adipose tissue. This amplification effect also made the relationship between the lesion and the surrounding organs more accurate, thus making the judgment of whether the lesion has invaded the peritoneum, spleen, liver and other organs more credible. One lesion in a patient with T4b who was examined using HR MRI showed that the lesion had invaded the left crus diaphragm, spleen and left lobe of the liver. The invasion of the left crus diaphragm and spleen was accurately diagnosed using this technique. Several studies have shown that combining T2WI, DWI and contrast-enhanced T1WI protocols could improve the accuracy of T staging of patients with gastric cancer, especially advanced gastric cancer [25, 29, 33, 34]. For patients who are not suitable for the use of contrast agents, DWI can even be used as an alternative for evaluating T staging [25]. With improved spatial resolution and image contrast, high-resolution diffusion-weighted and contrast-enhanced T1W images can accurately evaluate the depth of infiltration [22]. Therefore, combining HR MRI protocols has preferable diagnostic efficacy in evaluating the T stage in patients with esophagogastric junctional cancer.

According to the recently published National Comprehensive Cancer Network (NCCN) guidelines, patients with different T stages of esophagogastric junctional cancer should be approached with different treatment strategies [4]. For the treatment of patients with early T stage cancers, such as stages Tis, T1a and T1b, endoscopic resection and endoscopic ablation are preferred. Patients with stage T2 or T3 stages may have a chance of undergoing radical surgery or need neoadjuvant chemoradiotherapy before surgery. Most stage T4a patients need preoperative radiotherapy or chemotherapy, while stage T4b patients should receive only palliative treatment. Therefore, accurate screening of lesions below stage T1 and above stage T4 has a decisive significance for determining the treatment plan of patients [4].

For patients with a T stage ≥ T4, our study shows that the accuracy, sensitivity, specificity, overestimation, and underestimation of HR MRI were 93.5, 94.3, 88.9, 4.8 and 1.6%, respectively. However, no significant differences were observed between high-resolution and conventional MRI techniques in these parameters. For patients with a T stage of ≤ T1, the accuracy, sensitivity, specificity and overestimation of high-definition MRI were 98.4, 90, 100, 1.6 and 1.6%, respectively. The accuracy, specificity and underestimation rate of HR MRI were superior to those of conventional MRI; the sensitivity and overestimation rates were slightly different between these two techniques, and there were no significant differences in each parameter. One study reported no significant differences in the T staging of patients with ≤ T1 or ≥ T4 when using conventional multicontrast MRI protocols [25]. Another study reported that no significant differences were observed in the T stages of patients with ≥ T4 among conventional MRI without DWI, conventional MRI with DWI, and MDCT techniques [29]. In the present study, there were no significant differences in the T staging results between the two imaging techniques, which may have been due to an insufficient number of patients. There were 10 and 11 patients with ≤ T1 and 9 and 9 cases with ≥ T4 based on high-resolution and conventional MRI techniques, respectively.

There are several shortcomings in this study. First, the number of patients in each stage was small, and a comparison between different stages was not performed. Second, most of the patients had a staging of T3, while the number of patients with stages T1, T2 and T4 was relatively small, which may have led to selection bias. Third, although all patients underwent gastric water filling before the examination, different filling levels were observed because of the tolerance and compliance of each patient. Fourth, due to its smaller FOV, the use of HR MRI may lead to the loss of some key information, such as liver metastasis and retroperitoneal lymph node metastasis. Finally, all of the multicontrast protocols were used together for the evaluations, but the value of each sequence was not evaluated separately.

## Conclusions

In conclusion, HR MRI has a good diagnostic performance and may serve as an alternative technique in the T staging of patients with esophagogastric junction cancer in clinical practice.

## Data Availability

The datasets used and/or analyzed during the current study are available from the corresponding author on reasonable request.

## Abbreviations

AJCC: American Joint Committee on Cancer
DWI: Diffusion-weighted imaging
EUS: Endoscopic ultrasonography
FA: Flip angle
FOV: Field of view
FS: Fat saturation
ICC: Intraclass correlation coefficient
MDCT: Multi-detector spiral CT
MRI: Magnetic resonance imaging
NCCN: National Comprehensive Cancer Network
PET/CT: Positron emission tomography/computed tomography
ST: Slice thickness
TA: Acquisition time
TR/TE: Repetition time/echo time
TSE: Turbo spin echo
VIBE: Volumetric interpolated breath-hold examination
